# Clinical follow-up of 2 families with glomerulopathy caused by *COQ8B* gene variants and literature review

**DOI:** 10.3389/fped.2024.1378083

**Published:** 2025-01-14

**Authors:** Lei Zhang, Gentzon Hall, Peitong Han, Chunzhen Li, Jieyuan Cui

**Affiliations:** ^1^ Department of Pediatric Nephrology, Children's Hospital of Hebei Province Affiliated to Hebei Medical University, Shijiazhuang, China; ^2^ Duke University School of Medicine, Duke Molecular Physiology Institute, Durham, NC, United States

**Keywords:** *COQ8B* variant, glomerulopathy, steroid-resistant nephrotic syndrome, focal segmental glomerulosclerosis, autosomal recessive

## Abstract

**Background:**

Primary coenzyme Q10 (CoQ10) deficiency is an autosomal recessive genetic disease caused by mitochondrial dysfunction. Variants in Coenzyme Q8B (*COQ8B*) can cause primary CoQ10 deficiency. *COQ8B*-related glomerulopathy is a recently recognized glomerular disease that most often presents as steroid-resistant nephrotic syndrome (SRNS) in childhood. The disease often progresses to kidney failure and the renal histopathology is most commonly focal segmental glomerulosclerosis (FSGS).

**Methods:**

Four SRNS cases (2 females and 2 males) from 2 unrelated families who were followed clinically for nearly 3 years. Clinical exome testing and analyses were performed by MyGenostics Laboratory in China to evaluate unexplained proteinuria given the strong family history of glomerular disease and histologic evidence of SRNS. Pathogenic variants were identified in *COQ8B* in the exome studies and confirmed by direct sequencing.

**Results:**

Clinical exome sequencing revealed biallelic variants of the *COQ8B* gene in 2 families. In the Family 1, the oldest of three affected siblings died of renal failure at 11 years of age. Based on the results of genetic testing which identified a homozygous variant of *COQ8B*, the other two affected siblings with mild proteinuria and normal renal function were treated with CoQ10 oral supplementation at an early stage. Coenzyme Q10 treatment was effective in reducing proteinuria levels in both patients from Family 1 over the first 6 months and the two patients still have low-level proteinuria and normal renal function at nearly three years. In Family 2, clinical exome sequencing revealed a compoundheterozygous variants of *COQ8B* in a patient with biopsy- proven FSGS. His disease was unresponsive to prior treatment with glucocorticoids and cyclosporine. Oral CoQ10 was initiated based on his genetic diagnosis and was it was effective in reducing proteinuria over the first 5 months months of therapy. However after 1 year, his disease progressed tokidney failure. Kidney transplantation was performed at 5 years of age and his condition has been stable without rejection and no recurrence of disease.

**Conclusions:**

*COQ8B* gene variant-related glomerulopathy often presents as SRNS without obvious extrarenal manifestations. The histopathology is mainly FSGS and follows an autosomal recessive mode of inheritance. Some patients may benefit from early coenzyme Q10 supplementation. For patients whose disease progresses to kidney failure, kidney transplantation can be an effective treatment. For children with unexplained proteinuria and abnormal renal function, genetic testing should be performed early in the course of disease to guide therapy where possible and improve prognosis.

## Introduction

Primary coenzyme Q10 (CoQ10) deficiency is a heterogeneous genetic disorder caused by mitochondrial dysfunction. It is an autosomal recessive disease that most commonly manifests in childhood and may involve the nervous system, kidney, skeletal muscle and heart. When the kidney is involved, the typical presentation is steroid-resistant nephrotic syndrome (SRNS).

CoQ10 is essential for the proper functioning of the mitochondrial respiratory chain. CoQ10 is essential for cellular metabolism and energetics with crucial roles in ATP production, pyrimidine biosynthesis and the regulation of apoptosis. There are limited reports describing the pathogenic effects of *COQ8B* variants on CoQ biosynthesis and other related mitochondrial functions, especially in children with kidney disease. Insights from structural modeling suggest that pathogenic variants in *COQ8B* alter allosteric regulation or protein folding and stability in renal disease (1). A family of at least 17 different genes synthesize CoQ10 in mitochondria (2). COQ8-related genes include *COQ8A* (NM_020247.5) and *COQ8B* (NM_024876.4). A recent study showed that approximately 5.8% of SRNS patients had biallelic *COQ8B* variants. More than 26 variants have been reported in *COQ8B* alone according to the Human Gene Mutation Database (HGMD; https://www.hgmd.cf.ac.uk/ac/index.php). Clinically, *COQ8B* - related glomerulopathy presents as SRNS in childhood most commonly without associated extrarenal manifestations. FSGS is the most common histopathologic finding and the response to therapy can be heterogeneous. Here, we report the clinical features and therapeutic responses of 4 pediatric patients with glomerular disease due to *COQ8B* variants.

## Case presentations

A total of 4 patients (2 females and 2 males) from 2 unrelated families were included in this study.

### Family 1

**Figure 1:**
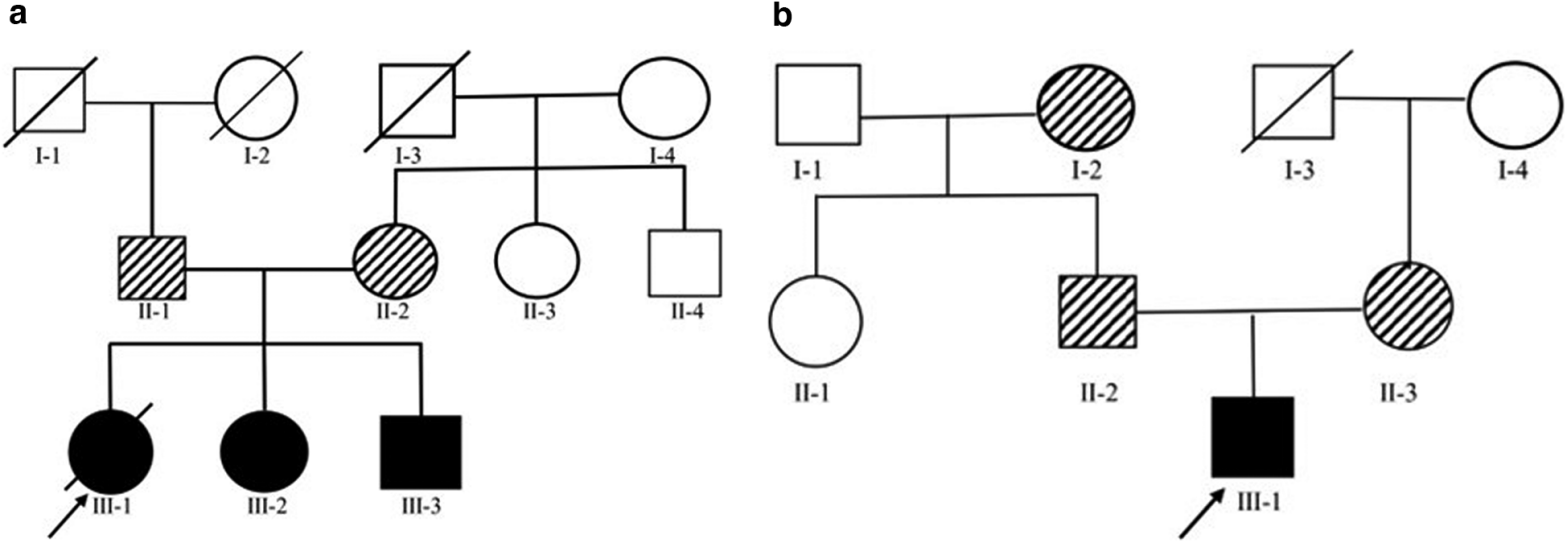
**(A)** Family 1 Pedigree - Cases 2 and 3 both carried homozygous variants of *COQ8B* with in exon 9 (c.737G>A) while both parents heterozygously carried the same variant. **(B)** Family 2 Pedigree - Case 4 carried two heterozygous variants, c.737G>A (a) and c.1465C>T (d) in *COQ8B*. While his mother and father heterozygously carried c.737G>A (c) and c.1465C>T (e), respectively.

**Figure 2:**
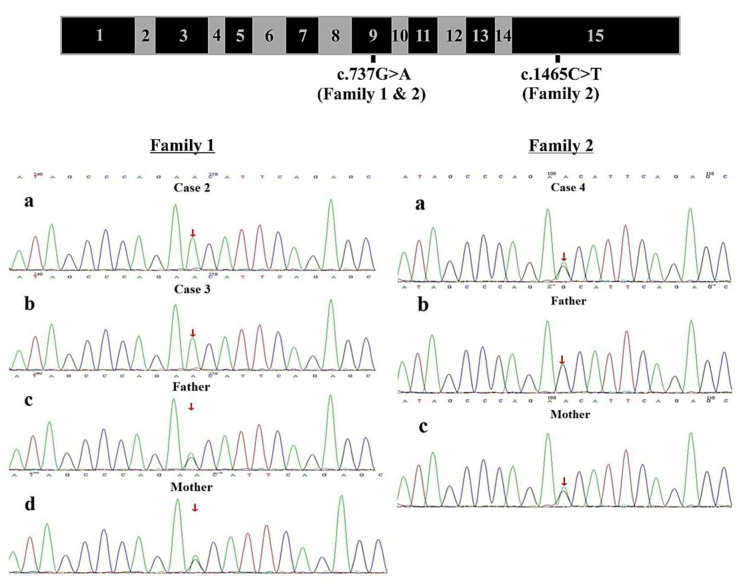
Genomic Structure of *COQ8B* and Family Sequencing -*COQ8B* gene structure with labeling of family variants in exons 9 and 15. Direct sequencing data for Family 1 showing 2 affected siblings with the homozygous *COQ8B* variant (c.737G>A) and both unaffected parents with a heterozygous *COQ8B* variants. Direct sequencing data for Family 2 showing 1 affected individual with a compound heterozygous variants (c.737G>A and c.1465C>T) of *COQ8B* and each unaffected parent with a single heterozygous variant.

The oldest of three affected siblings from a 3-generation kindred was diagnosed with non-syndromic proteinuric kidney disease at age 9-year old (Figure 1a). She died two years later from kidney failure and had not received CoQ10. Her two surviving siblings who had proteinuria and normal renal function, underwent clinical exome sequencing and genotyped for a homozygous COQ8B variant (c.737G>A) (Figure 2a). The family denies a history of consanquinity and no we were not able to identify evidence of consanguineous relationships through our analyses. Based on these findings, oral CoQ10 therapy was initiated. CoQ10 treatment was effective in reducing proteinuria over the first six months of therapy and the siblings have retained normal renal function (Table 1).

**Table 1:** Clinical data for the families 1 & 2.

Items	Case 1	Case 2	Case 3	Case 4
Family	1	1	1	2
Gender	Female	Female	Male	Male
Ethnicity	Chinese	Chinese	Chinese	Chinese
Onset age (year)	9	8	5	3
Proteinuria before CoQ10 Tr^a^	–	1.18	0.78	1.62
(g/24 h) after CoQ10 Tr^a^	–	0.22–0.32	0.11–0.18	0.33
Serum albumin (g/L)		39.5	35.7	40.3
Serum creatinine (µmol/L)	346.3	34.0	42.0	31.0
Renal pathology	–	–	–	FSGS
CoQ10 dosage (mg/kg/day)	–	100	100	100
Follow-up time (year)	2.4	2	2	3.5
CKD stage	5	1	1	5
Age at death (year)	11	–	–	–
COQ8B variants	c.737G>A (p.S246N), hom^a^			c.737G>A (p.S246N), het^a^ c.1465C>T (p.H489Y), het^a^

^a^ Tr, treatment; hom, homozygous; het, heterozygous.

### Family 2

A fourth individual was diagnosed with FSGS (Figure 1b). The patient was initially treated with glucocorticoids and cyclosporine without response. Clinical exome analyses revealed a pathogenic compound heterozygous variant of *COQ8B* (c.737G>A and c.1465C>T) (Figure 1b). Based on this finding, oral CoQ10 therapy was initiated. CoQ10 was effective in reducing his proteinuria over the first ∼4 months of therapy, however, his renal function began to deteriorate after 1 year of treatment and he progressed to kidney failure (Table 1). Kidney transplantation wassubsequently performed and his condition stabilized with no recurrence of disease.

Parents of both families were healthy, with non-consanguineous marriages or family history of kidney disease. After obtaining informed consent from all parents, peripheral blood was collected from patients and their respective parents for genetic analyses. Clinical exome testing and analyses were performed by MyGenostics Laboratory in China. Results showed a homozygous variant c.737G>A (p.S246N) (PM3) of *COQ8B* gene within exon 9 which was reported in The Human Gene Mutation Database (HGMD; https://www.hgmd.cf.ac.uk/ac/index.php). Both parents were heterozygous for the variant (Figure 2). The expected segregation of putative variants was confirmed in families, whenever possible, and their absence was confirmed in SNPs databases of common benign variants (http://www.ncbi.nlm.nih.gov/projects/SNP/ and www.gnomad-sg.org). Human Splicing Finder (http://www.umd.be/HSF/) and Mutation Taster (http://mutationtaster.org/) were used for pathogenicity prediction of the variants, respectively andthe c.737G>A (p.S246N) variant were predicted to be “damaging” or “possibly damaging”.

**Figure 3:**
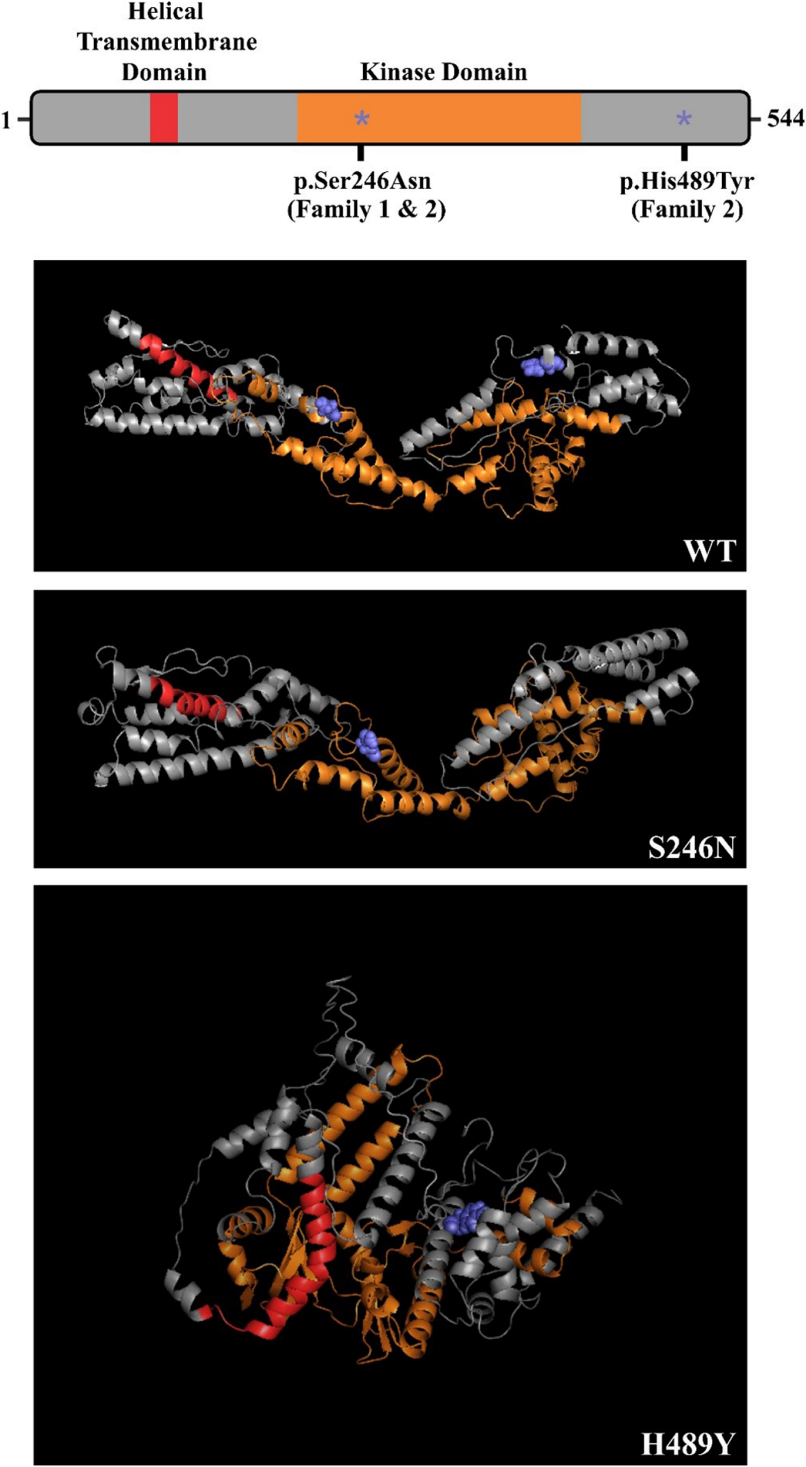
Predicted effect of family COQ8B variants on protein secondary structure – COQ8B domain structure depicted with p.S246N and p.H489Y variants labeled (*). Both variants are predicted to disrupt the secondary structure of the protein.

In Family 2, the exome analysis of the male proband showed two heterozygous variants c.737G>A (p.S246N) and c.1465C>T (p.H489Y), in exon 9 and exon 15 of *COQ8B* gene, respectively (Figure 2). His mother carried the c.737G>A variant while his father carried the c.1465C>T variant (Figure 2). The rare c.737G>A variant was found in the SNP databases (rs200841458, A = 0./0, 0.000043/6 and 0.000057/15 in ALFA, GnomAD and TOPMED, both heterozygotes, respectively)with a minor allele frequency of 0.000037 and was found almost exclusively in individuals of East Asian ancestry. The c.1465C>T (p. H489Y) variant was also found in the SNP databases (rs139063940, A = 0./0, 0.000013/3 and 0.000008/2 in ALFA, GnomAD and TOPMED, both heterozygotes, respectively),was rare with a minor allele frequency of 0.0000037, and was almost exclusively expressed in individuals of East Asian ancestry. PolyPhen-2, SIFT, Variant Taster and GERP++ analysis predicted that both the two variants were “damaging” or “possibly damaging”. Additionally, both the p.S246N and the p.H489Y variants are predicted to disrupt the secondary structure of the *COQ8B* protein (Figure 3).

After diagnosis was confirmed as *COQ8B*-related glomerulopathy, cases 2, 3 and 4 were treated immediately with oral supplementation of CoQ10 (100 mg/kg/day). In case 2 from Family 1, her appetite improved, weight began to increase, and urine protein began to decrease after the first month of treatment. Her weight increased by 3 kg and her total 24-h urine protein decreased to 0.27 g after 3 months. Her total 24-h urine protein remained between 0.22 and 0.32 g, and her renal function remained normal over 2 years of follow up.

In case 3 from Family 1, the total 24-h urine protein decreased to 0.13 g after 3 months and remained between 0.11 and 0.18 g over 2 years follow up. His renal function has remained normal.

**Figure 4:**
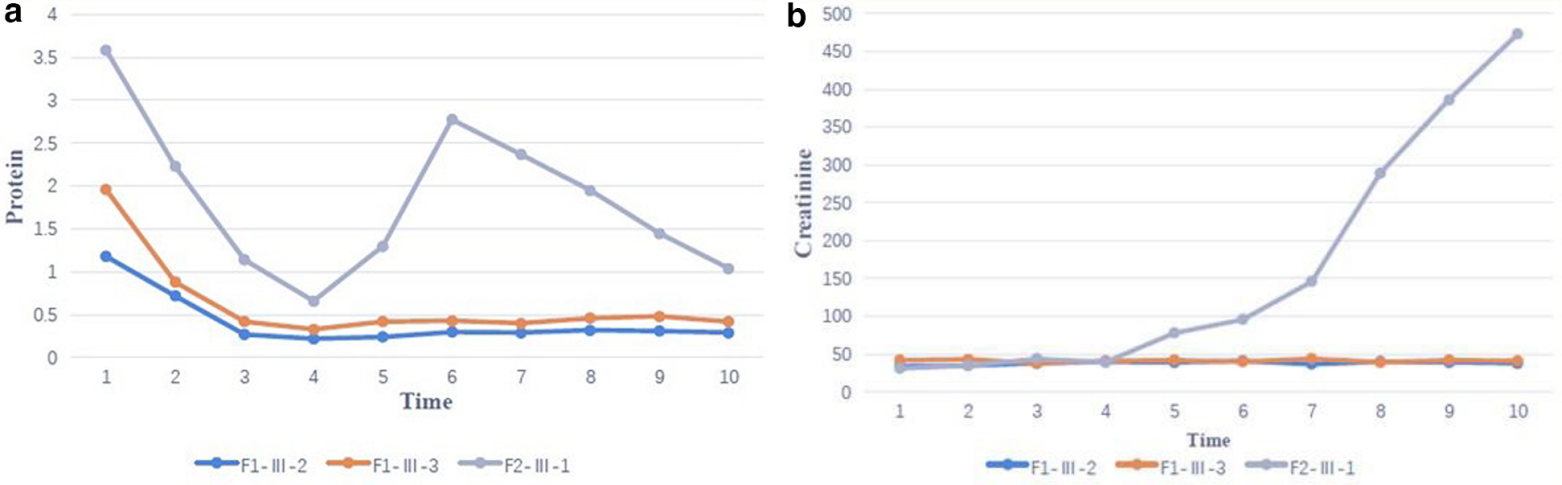
**(a)** Family proteinuria response trajectories - change in proteinuria during coenzyme Q10 (CoQ10) supplementation over 2 years of follow-up (F1-Ⅲ-2, F1-Ⅲ-3 and F2-Ⅲ-1 correspond to case 2,3 and 4). **(b)** Family eGFR Response Trajectories – Change in serum creatinine during coenzyme Q10 (CoQ10) supplementation over 2 years of follow-up (F1-Ⅲ-2, F1-Ⅲ-3 and F2-Ⅲ-1 correspond to case 2,3 and 4).

**Figure 5:**
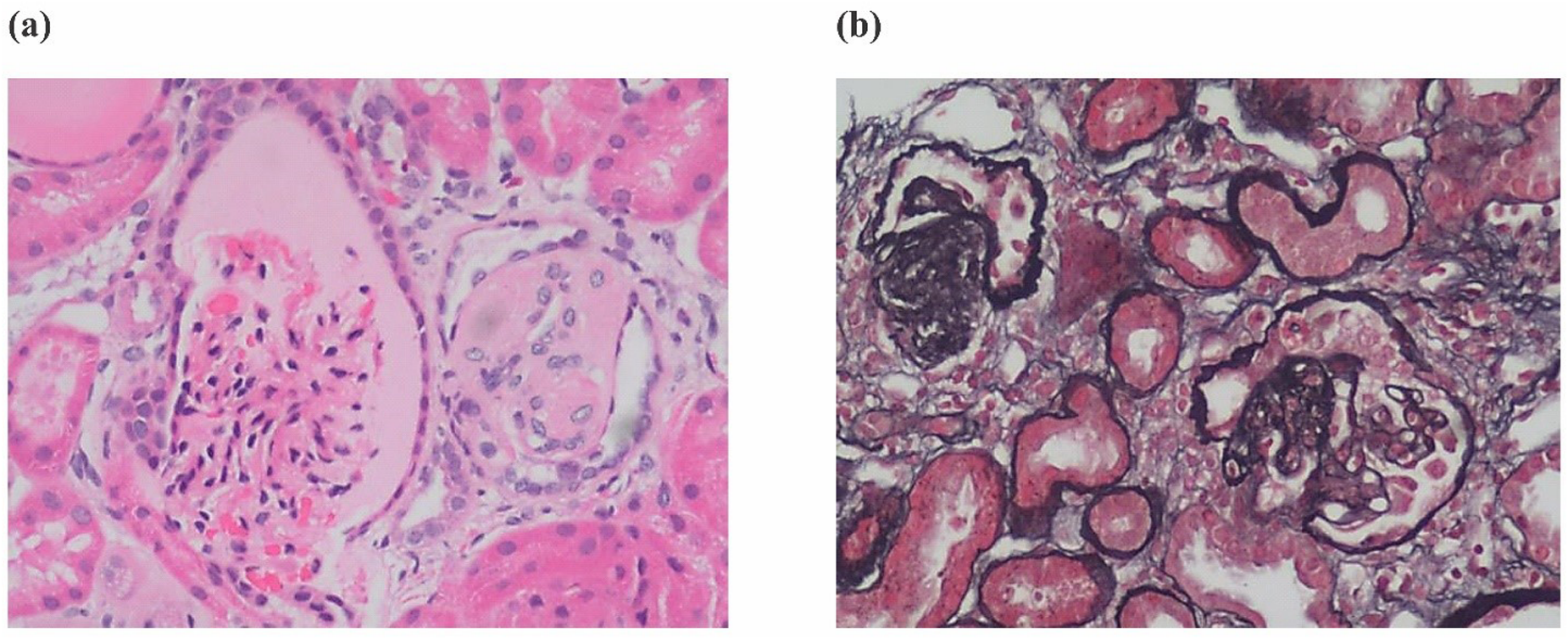
Renal histopathology - **(a)** renal histopathology of case 4 showing FSGS by H&E staining and **(b)** Silver Jones Methenamine staining.

In case 4 from Family 2, his total 24-h urine protein decreased to 0.33 g after ∼4 months of CoQ10 supplementation. However, his proteinuria and serum creatinine level began to increase after 1 year (Figures 4a,b). His kidney biopsy showed FSGS (Figure 5). Under light microscopy, Case 4 shows 43 glomeruli, 21 of which were sclerotic, and vacuolar degeneration was observed in the renal tubules. Electron microscopy findings were consistent with the light microscopy and no mitochondrial morphological abnormalities were detected. He reached CKD stage 5 two years later and peritoneal dialysis was initiated. He underwent kidney transplantation after 2.5 years. He has experienced no episodes of rejection and no recurrence of disease post-transplant. During the entire treatment period, the child had no severe or recurrent infections and was not exposed to any nephrotoxic drugs.

## Discussion

Pathogenic *COQ8B* variants can cause primary CoQ10 deficiency; a clinically and genetically heterogeneous disorder. *COQ8B* gene variant-related glomerulopathy is a recently recognized glomerular disease associated with coenzyme Q deficiency that most commonly manifests in early childhood as SRNS with FSGS histopathology that can progress to kidney failure (1, 2). SRNS is one of the leading causes of kidney failure in children and young adults, and its renal histopathology is most commonly FSGS (3, 4). Reports suggest that *COQ8B*-related glomerulopathy is a progressive disease and an important consideration in the differential diagnosis for adolescent kidney failure patients (3–6). CoQ10 deficiency often affects multiple systems, including the nervous system, kidneys, skeletal muscle, and heart. Reports of extrarenal manifestations in children with *COQ8B*-related glomerulopathy are rare (7). A total of 4 patients (2 females and 2 males) from 2 unrelated families were evaluated in this study. In both families, neither of the patients had extrarenal manifestations of disease and all affected individuals presented with isolated proteinuria with or without renal dysfunction. This report is unique because there have been no cases previously reported in the Han Chinese subpopulation in northern China. Additionally, the cases highlighted in this report delineate the treatment outcomes of three children who received similar care after ∼3 years of follow up. As with other cases, the reason for the heterogeneity of the treatment responses in these families is not clear. This re-emphasizes the idiosyncratic nature of genetic kidney disease and the urgent need for larger randomized controlled trials of diagnosis and therapy for monogenic *COQ8B*-associated nephropathy to establish optimal treatment algorithms for the disease and to define at-risk populations. Such studies are also needed to provide more precise frequency estimates of the burden of disease. In 2018, Wang et al. showed that *CO8QB* variants accounted for the 6.67% of monogenic SRNS cases in a large Chinese cohort (8). *CO8QB* variants were the most common cause of monogenic SRNS in this population suggesting a unique ancestry-driven predisposition and highlighting a need for early genetic testing in an at-risk population.

FSGS was the most common pathological finding on renal biopsy in *COQ8B*-related glomerulopathy (7). In this study, the renal histopathology of Case 4 was FSGS while the other 3 cases did not undergo renal biopsy. The histopathology of *COQ8B*-related glomerulopathy lacks pathognomonic morphological features, and the specific mechanism of its predisposition to FSGS is unknown. *COQ8B* is expressed in mitochondria of podocytes, proximal tubules and collecting ducts. There are no characteristic features of CoQ10 deficiency by light other than the FSGS lesion, however, several reports have documented abnormal tubular cell morphology characterized as granular swollen epithelial cells (GSECs). Therefore, identifying GESCs by light microscopy may be useful for identification of *COQ8B* nephropathy and other CoQ10 deficiencies (9). Renal histopathology of some patients manifested as mild glomerular abnormalities by light microscopy, together with mitochondrial proliferation, hypertrophy and crowding by electron microscopy (10). *COQ8B* glomerulopathy often does not respond to corticosteroid and immunosuppressive therapy. Fortunately, some patients benefit from early coenzyme Q10 supplementation. In our study, the oldest affected sibling (Case 1) from Family 1 was diagnosed with kidney failure at the age of 9 and died at 11 years old. No genetic diagnosis was established and she did not receive CoQ10. Coenzyme Q10 treatment was effective in reducing proteinuria and appeared to be protective in the other 3 cases, with a significant reduction in urinary protein after about half a year of treatment. Unfortunately, proteinuria began to increase after 1 year of follow-up in this patient as his disease progressed. The therapeutic effect of coenzyme Q10 supplementation is not uniform in patients with renal disease associated with coenzyme Q deficiency. This may due to a variety of factors such as delay in the onset of therapy, variability in disease chronicity, severity of the disease-causing genetic variant, additive or synergistic intergenic interactions, etc. Documented responses vary from no response, partial remission, or complete disappearance of urinary protein (4, 11, 12). Efficacy of supplementation of exogeneous CoQ10 has been shown in some patients with CoQ10 nephropathies and the rare side effects of treatment were mild (13). Initiating exogenous coenzyme Q10 (CoQ10) supplementation early in the asymptomatic period has been shown to reduce total urinary protein and appears to have a protective effect on renal function in some children (14). Other studies have demonstrated that CoQ10 supplementation led to significantly improved preservation of kidney function (15). Despite these cases of treatment success, other studies have shown less favorable outcomes suggesting that successful treatment may depend on a diversity of factors (i.e., genetic diagnosis, timing of therapy initiation, comorbid disease, etc.) (16). The reason for the non-uniform therapeutic effect of coenzyme Q10 supplementation remains unclear and there is no genotype-phenotype correlation described so far. Notably, there are no studies addressing whether supplementation of CoQ10 has a reno-protective potential if started in an asymptomatic period before irreversible renal damage is established. There are also no studies on the specific dose and duration of oral supplementation with CoQ10. Most physicians still recommend early application and life-long treatment. It has also been suggested that early detection of *COQ8B* nephropathy after supplementation with CoQ10 in combination with ACE inhibitors can slow the progression of renal insufficiency. Kidney tranplantation in patients with familial SRNS, such as COQ8-associated nephropathy, was not associated with recurrence of the underlying diseas (17). The commonly used dose is 100 mg/kg/day, which was also adopted in our treatment strategy. Of our 3 patients who received exogenous COQ10, 2 responded well to this regimen and their renal function remains normal to date. However, in case 4, renal function initially improved with treatment, but began to deteriorate 1 year later, and eventually progressed to kidney failure. This is consistent with other cases reported in the literature (18). Therefore, the protective effect of exogenous COQ10 on renal function still needs further observation and research, which may be affected by rarity of the disease. In summary, genetic testing, including investigation of genes involved in CoQ10 biosynthesis, should be considered in young patients with proteinuria of unknown etiology or CKD, especially without extrarenal manifestations. Early initiation of therapy may reduce proteinuria and preserve kidney function.

## Conclusion

*COQ8B*-associated glomerulopathy often presents as SRNS with no obvious extrarenal manifestations. It has been identified as a significant cause of monogenic nephropathy in some populations and exhibits a variable response to CoQ10. The histopathology is predominantly FSGS, and there are no pathognomonic morphological features of the disease. Patients often do not respond to corticosteroid or immunosuppressive therapy, however, a considerable number of children may respond to early supplementation with coenzyme Q10 therapy. In these patients, CoQ10 can reduce proteinuria and may protect kidney function. Unfortunately, some patients will still develop to kidney failure. For these individuals, kidney transplantation can an effective treatment option. For children with unexplained proteinuria and abnormal renal function, genetic testing should be performed early in the course of disease as a diagnosis of *COQ8B*-glomerulopathy may be treatable.

We acknowledge the limitations of this concise report, and we will pursue further studies in a larger cohort in the future. Due to the limited sample size, our study results may not be generalizable to the Han Chinese population. To address these limitations, a broader evaluation of familial SRNS cases in this population is needed to better characterize the epidemiology of *COQ8B*-associated nephropathy and the efficacy of CoQ10 therapy. None the less, our findings are consistent with prior reports in other subgroups and identify the Han Chinese as an at-risk population for the SRNS caused by pathogenic *COQ8B* variants.

## Data Availability

The original contributions presented in the study are included in the article/supplementary materials, further inquiries can be directed to the corresponding authors.
